# Supplementary material for: Screening of Multitarget Compounds against Acetaminophen Hepatic Toxicity Using In Silico, In Vitro, and In Vivo Approaches
Source: Molecules. 2024 Jan 16;29(2):428. doi: 10.3390/molecules29020428 (PMC10821416; doi:10.3390/molecules29020428)
Supplement: Supplementary file 1 [file molecules-29-00428-s001.zip › molecules-2713918-supplementary.pdf]

Supplementary Materials:

Table S1. List of compounds selected for docking analysis from each plant.

C. longa	
(+)-Alpha-Curcumene	(-)-Alpha-Cedrene
Ar-Turmerone	BETA-ELEMENE
Curcumenol	Beta-Elemenone
Beta-Vetivenene	Gamma-Curcumene
Calebin-A	Beta-Sesquiphellandrene
Curcumin	Beta-Curcumene
Bisdemethoxycurcumin	1-(4-Hydroxy-3,5-Dimethoxyphenyl)
Demethoxycurcumin	2-Methoxy-4-Vinylphenol
Vanillin	Vanillic Acid
(3Z,7Z)-3,7-dimethyl-10-propan-2-ylidenecyclodeca-3,7-dien-1-one	6R)-2-methyl-6-[(1R)-4-methylidenecyclohex-2-en-1-yl]hept-2-en-4-one
C. zeylanicum	
RUTIN	Cis-P-Coumaric Acid
Acarbose	Cinnamate
Quercetin	Eugenol
Gallic Acid	CINNAMIC ACID
SINAPIC ACID	BENZYL BENZOATE
3,4-DIHYDROXYBENZOIC ACID	Eugenol Acetate
Vanillic Acid	Cinnamyl Acetate
Trans-2-Hydroxycinnamic Acid	Cinnamaldehyde
(-)-Linalool	D-CAMPHOR
(-)-Endo-Alpha-Bergamotene	(1R,9S)-4,11,11-trimethyl-8-methylidenebicyclo[7.2.0]undec-4-ene

Table S2. Property profile of top five selected compounds from *C. longa* plant against two selected receptors.

Sr.	Ligand	Receptor/Enzymes	PDB Code	S-Score	RMSD
1	Curcumin	CYP2E1	3e6i	-14.3	0.99
2	Calebin	CYP2E1	3e6i	-14.1	1.60
3	Demethoxycurcmin	CYP2E1	3e6i	-13.4	0.82
4	1-(4-Hydroxy-3,5-Dimethoxyphenyl)	CYP2E1	3e6i	-13.3	0.77
5	Bisdemethoxycurcumin	CYP2E1	3e6i	-12.7	1.07
6	1-(4-Hydroxy-3,5-Dimethoxyphenyl)	TLR2	1FYW	-12.5	3.00
7	Calebin	TLR2	1FYW	-12.3	1.28
8	Demethoxycurcmin	TLR2	1FYW	-11.9	0.57
9	Bisdemethoxycurcumin	TLR2	1FYW	-11.8	2.92
10	Curcumin	TLR2	1FYW	-10.7	1.09

Table S3. Property profile of top five selected compounds from *C. zeylanicum* plant against two selected receptors.

Sr. No.	Ligand	Receptor/Enzymes	PDB Code	S-Score	RMSD
1	RUTIN	CYP2E1	3e6i	-18.9	1.78
2	Acarbose	CYP2E1	3e6i	-15.7	1.45

3	Quercetin	CYP2E1	3e6i	-14.0	1.71
4	Trans-2-Hydroxycinnamic Acid	CYP2E1	3e6i	-11.8	0.93
5	SINAPIC ACID	CYP2E1	3e6i	-10.4	0.71
6	RUTIN	TLR2	1FYW	-16.2	1.77
7	Acarbose	TLR2	1FYW	-15.1	3.33
8	Quercetin	TLR2	1FYW	-14.6	1.12
9	Gallic Acid	TLR2	1FYW	-10.9	0.86
10	SINAPIC ACID	TLR2	1FYW	-10.0	1.92

Table S4. Absorption, metabolism, and toxicity-related drug-like properties of the best-chosen compound are shown using ADMET profiling.

	Curcumin	Quercetin	Caliben A	Rutin
Absorption				
BBB	-	-	-	-
HIA	+	+	+	+
CaCo2 permeability	-	-	-	-
PGS	-	-	-	-
PGI	+	-	-	-
ROCT	-	-	-	-
Metabolism				
CYP3A4 substrate	-	+	-	+
CYP2C9 substrate	-	-	-	-
CYP2D6 substrate	-	-	-	-
CYP3A4 inhibition	-	+	-	-
CYP2C9 inhibition	+	-	+	-
CYP2C19inhibition	+	-	+	-
CYP2D6 inhibition	+	-	-	-
CYP1A2 inhibition	+	+	+	-
Toxicity				
Ames toxicity	-	+	-	-
Carcinogens	-	-	-	+

Following are abbreviations including ROCT for renal organic cation transporter; HIA: human intestinal absorption; PGS: P-glycoprotein substrate; PGI: P-glycoprotein inhibitor; BBB for blood-brain barrier.

Table S5. S-score table of curcumin, quercetin and silymarin with liver injury receptors.

Sr. No	Receptor/Enzymes	Ligand	S-score
	TLR4	Curcumin	-7.3
	NF-kB	Curcumin	-6.0
	NLRP3	Curcumin	-5.8
	MAPKs	Curcumin	-5.8
	CYP1A2	Curcumin	-8.7
	CYP2C9	Curcumin	-9.2
	CYP2E1	Curcumin	-6.1
	CYP2D6	Curcumin	-7.9
	CYP3A4	Curcumin	-7.8
	COX-2	Curcumin	-6.0
	PINK-1	Curcumin	-6.0
	FXR	Curcumin	-5.7
	TGF-β	Curcumin	-4.9
	LRH-1	Curcumin	-4.2

RAGE	Curcumin	-6.3
TLR4	Quercetin	-8.2
NF-kB	Quercetin	-8.7
NLRP3	Quercetin	-7.5
MAPKs	Quercetin	-9.2
CYP1A2	Quercetin	-8.3
CYP2C9	Quercetin	-8.8
CYP2E1	Quercetin	-7.4
CYP2D6	Quercetin	-8.1
CYP3A4	Quercetin	-7.5
COX-2	Quercetin	-8.3
PINK-1	Quercetin	-7.2
FXR	Quercetin	-8.7
TGF-β	Quercetin	-7.1
LRH-1	Quercetin	-7.7
RAGE	Quercetin	-9.3
TLR4	Silymarin	-9.0
NF-kB	Silymarin	-8.7
NLRP3	Silymarin	-10.8
MAPKs	Silymarin	-10.8
CYP1A2	Silymarin	-9.8
CYP2C9	Silymarin	-8.5
CYP2E1	Silymarin	-8.9
CYP2D6	Silymarin	-9.0
CYP3A4	Silymarin	-9.8
COX-2	Silymarin	-8.9
PINK-1	Silymarin	-10
FXR	Silymarin	-9.8
TGF-β	Silymarin	-8.8
LRH-1	Silymarin	-9.2
RAGE	Silymarin	-10.7

Table S6. Interactions of curcumin, quercetin and silymarin with liver injury receptors/enzymes.

Receptor/Enzymes Name	Curcumin	Quercetin	Silymarin
TLR4	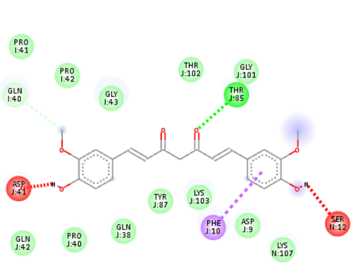	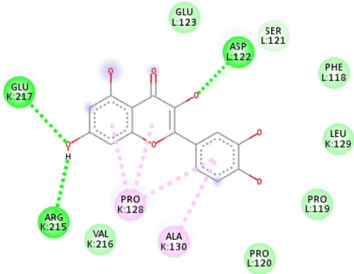	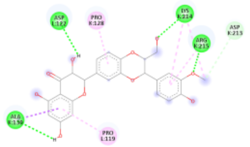
NF-kB	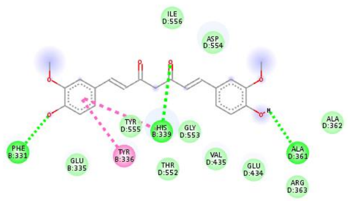	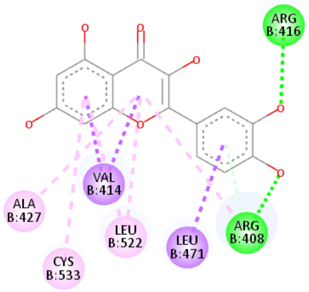	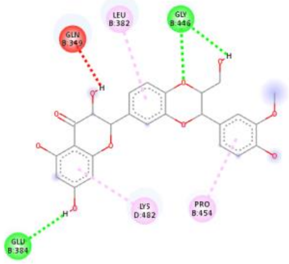
NLRP3	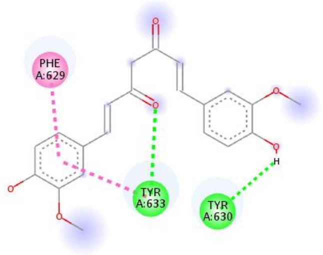	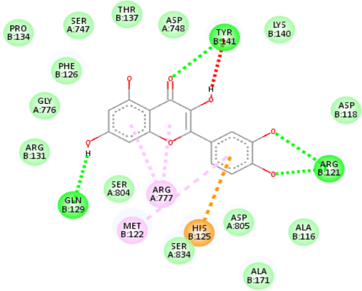	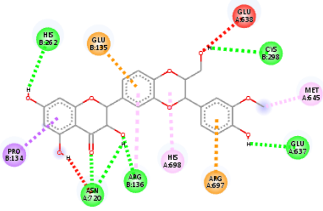
MAPKs	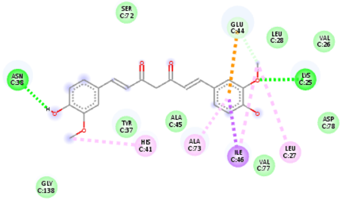	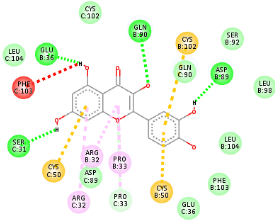	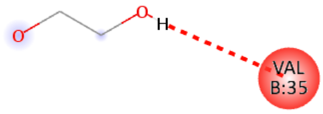
CYP1A2	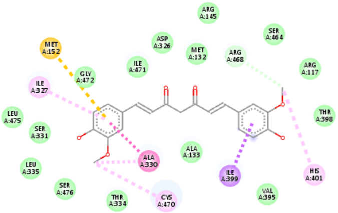	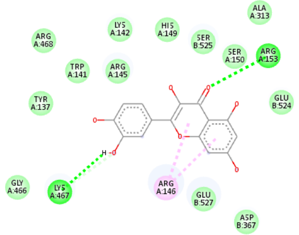	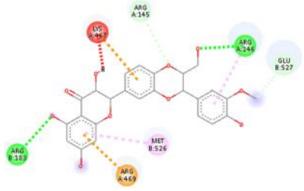

CYP2C9

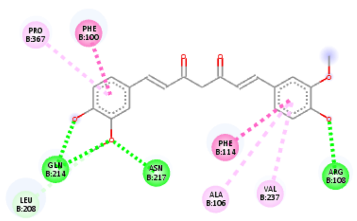

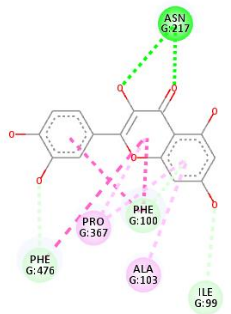

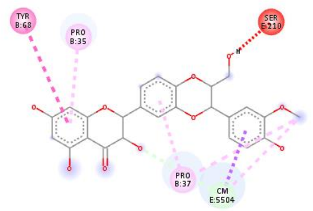

CYP2E1

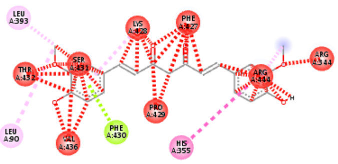

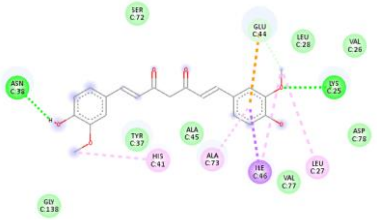

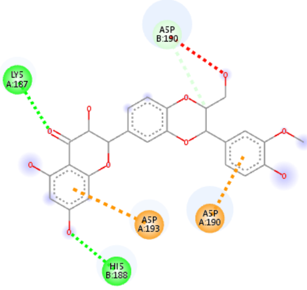

CYP2D6

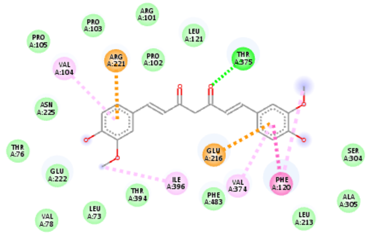

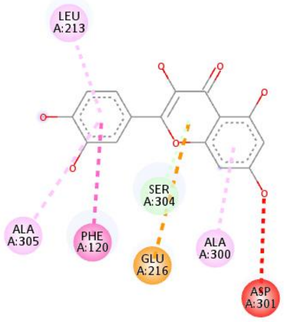

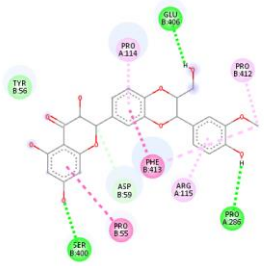

CYP3A4

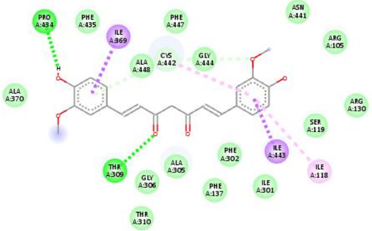

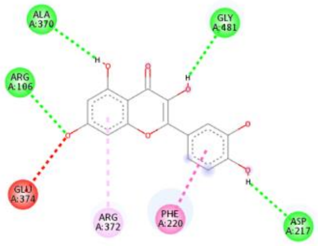

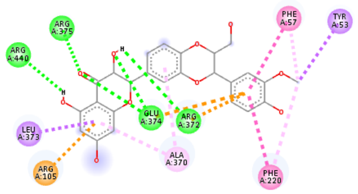

COX-2

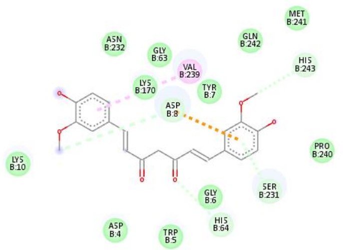

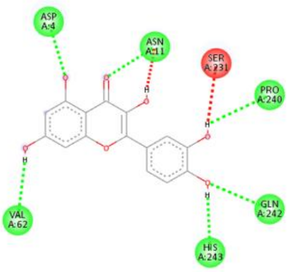

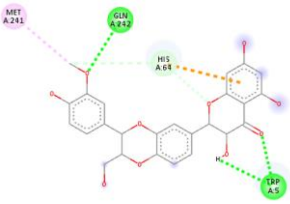

PINK-1

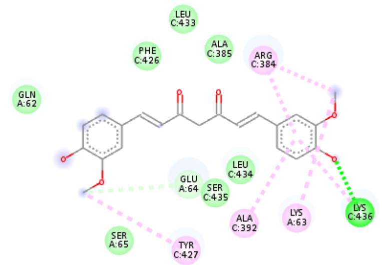

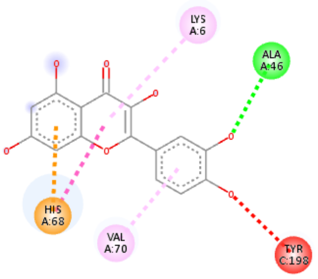

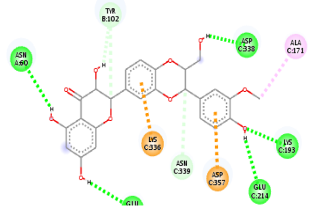

FXR

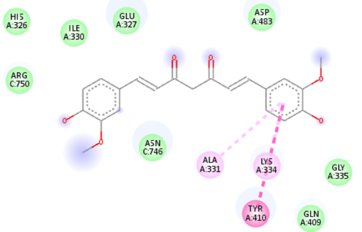

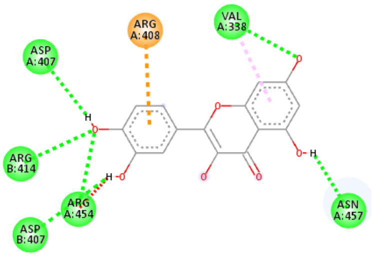

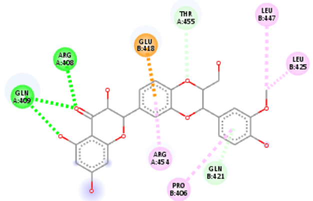

TGF-β

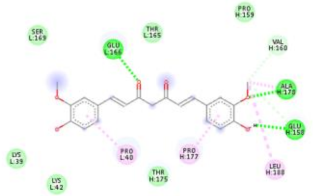

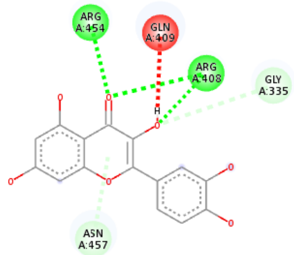

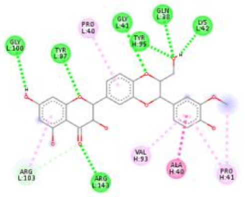

LRH-1

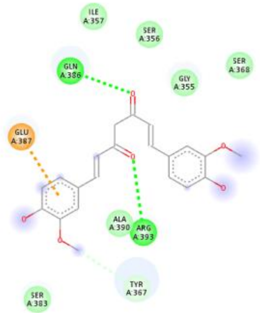

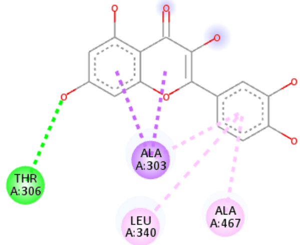

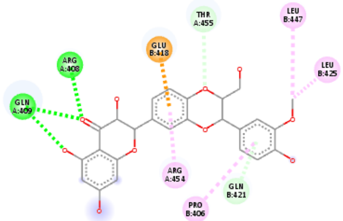

RAGE

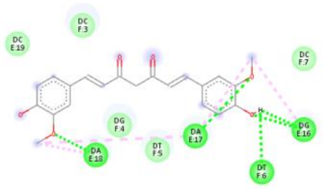

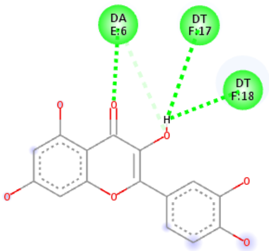

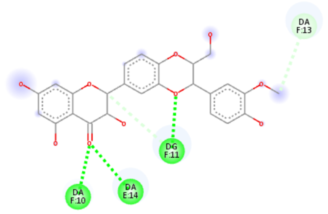

Table S7. Values of serum enzymes ALT, AST & ALP and serum bilirubin (T-Bil).

Treatment	ALT	AST	ALP	Bilirubin
Control group	116.33 ± 0.1	180 ± 0.01	126.05 ± 0.01	0.55 ± 0.1
Induced toxicity group	552 ± 0.01	287.67 ± 0.02	134 ± 0.1	0.52 ± 0.02
Protective group of Quercetin	75.5 ± 0.1	192.5 ± 0.1	27.5 ± 0.01	0.47 ± 0.1
Protective group of Curcumin	90.67 ± 0.02	90.67 ± 0.02	103.33 ± 0.02	0.51 ± 0.01
Protective group of combination	147.5 ± 0.1	176 ± 0.1	158 ± 0.1	0.57 ± 0.02
Protective group for silymarin	177.67 ± 0.02	223 ± 0.02	46 ± 0.01	0.45 ± 0.1

Results are expressed as mean ± (SEM).
